# Rationale and design of the Birkebeiner Ageing Study – a prospective cohort study of older endurance athletes

**DOI:** 10.1186/s12877-023-04099-3

**Published:** 2023-06-13

**Authors:** Marius Myrstad, Kristoffer Robin Johansen, Eivind Sørensen, Anette Hylen Ranhoff

**Affiliations:** 1 Department of Medical Research, Bærum Hospital, Vestre Viken Hospital Trust, N-1346 Gjettum, Norway; 2 Department of Internal Medicine, Bærum Hospital, Vestre Viken Hospital Trust, N-1346 Gjettum, Norway; 3 School of Sport Sciences, Faculty of Health Sciences, UiT The Arctic University of Norway, Tromsø, Norway; 4 Department of Internal Medicine, Diakonhjemmet Hospital, N-0370 Oslo, Norway; 5 Department of Clinical Science, University of Bergen, Bergen, Norway; 6 Norwegian Institute of Public Health, Oslo, Norway

**Keywords:** Athletes, Physical activity, Exercise, Aging

## Abstract

**Background:**

While regular physical activity is associated with reduced mortality and morbidity in general populations, health outcomes and functional capacity related to upholding strenuous endurance exercise beyond the age of 65 years are only sparsely studied. The aim of this study is to assess associations of prolonged strenuous endurance sport practice with ageing, functional decline, morbidity and longevity among older recreational endurance athletes, during long-term follow-up.

**Methods:**

Prospective cohort study of older recreational endurance athletes in Norway. All skiers aged 65 years and older who participated in a long-distance endurance competition, the annual 54-km Birkebeiner cross-country ski race in 2009 or 2010, were invited. The participants answered an extensive baseline questionnaire about lifestyle habits, including leisure-time physical activity and endurance sport participation, diseases, medication use and physical and mental health, with follow-up questionnaires planned every fifth year until 2029. New participants may be invited with the aim to increase the study size. Endpoints such as all-cause and disease-specific mortality, incidence and cumulative prevalence of diseases, use of medication, physical and mental health and functional decline will be assessed subsequently.

Out of 658 invited skiers (51 women), 551(84%) completed the baseline questionnaire and were included in the study. The mean age was 68.8 years (median 68, range 65- 90). At baseline, the participants had completed the Birkebeiner race for an average of 16.6 years and reported an average of 33.4 years of regular endurance exercise, with one out of five reporting at least 50 years of exercise. In all, 479 (90%) reported that they were still practicing leisure-time physical activity of moderate or vigorous intensity at least twice weekly. The prevalence of cardiovascular risk factors and diseases was low.

**Discussion:**

This prospective study of a cohort of recreational athletes exposed to prolonged and strenuous endurance exercise, could complement population-based studies by providing data on associations between life-long endurance sport participation, aging, functional decline and health outcomes during long-term follow-up.

## Background

Hippocrates suggested already 400 years B.C. that people with illness would profit from gymnastics and that healthy people would take advantage of exercise to maintain their health and well-being [1]. The role of physical activity (PA) and exercise in promoting healthy aging and preventing adverse health outcomes is not of less interest more than 2,000 years later [2].

While PA is often defined as any bodily movement resulting in energy expenditure, exercise or exercise training describe structured, repetitive activities that aim to improve or maintain physical fitness [3]. Moreover, endurance sports are performed at different levels of intensity, with varying demands to the cardiorespiratory system. PA and exercise are performed for short time periods, but also over many years and as part of an individual’s lifestyle. Thus, both PA and exercise are components of a continuum of exposure, and mounting evidence suggests that cumulative exposure may associate with health outcomes in a dose–response manner [4]. Regular PA has considerable beneficial effects on survival and risk of developing chronic age-related diseases [4–9], and the mortality benefit related to PA may be larger in older compared to younger individuals [10]. Regular PA at older ages also seems to prevent cognitive and physical functional decline and reduce the risk of dementia [11, 12]. Thus, PA may represent one of the most crucial components of healthy and successful aging. Consequently, recently published guidelines suggest that moderate PA should be performed for 150–300 min/week [13, 14].

Importantly, already limited amounts of PA far below the current guidelines may have beneficial health effects compared to physical inactivity [6]. In fact, the largest benefit in terms of reduced mortality and morbidity is seen when comparing low PA to inactivity [15, 16]. With increasing levels of PA, the benefits may be even larger, with a suggested plateau occurring around 300 min per week [7].

However, an unknown proportion of the population practice regular exercise beyond moderate PA [17]. Several studies indicate that participation in long-distance endurance sports competitions, such as marathon running, cycling and cross-country skiing, has increased over time, even among people aged 65 years and older [18, 19].

While the health benefits of leisure-time PA of low or moderate intensity are indisputable, effects on health outcomes related to more strenuous forms of PA, such as endurance exercise performed throughout years and decades, are less studied. A few prospective studies have reported that elite athletes have lower mortality compared to the general population [20–23]. Also, sports participation on recreational levels seems to be associated with reduced mortality [24, 25]. However, only a few studies have investigated how the highest levels of PA may associate with the risk of diseases and disabilities later in life, compared to less strenuous activities.

**Fig. 1:**
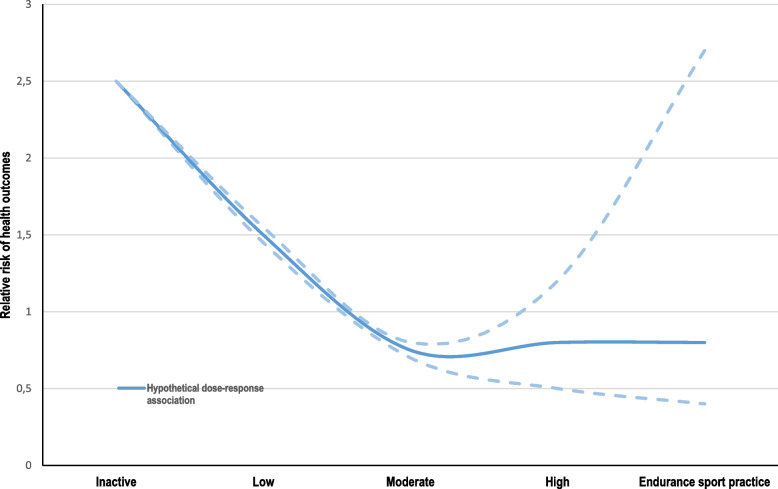
Relative risk of health outcomes at older ages and illustrative confidence intervals, related to different levels of leisure-time physical activity. While the health benefits of low to moderate physical activity are well documented, the effects of the highest level of physical activity and prolonged endurance sports practice are largely unknown

The shape of the association between prolonged and repeated exposure to high-intensity endurance sports and health outcomes remains largely unknown. While the association seems to be linear for some endpoints, studies have revealed an increased risk of cardiac arrhythmias related to vigorous and competitive endurance sport practice [26], suggesting a U-shaped dose–response relationship (Fig. 1).

The Copenhagen City study found the lowest cardiovascular and all-cause mortality among individuals who reported a weekly duration of sports activities of 2.6 to 4.5 h. Those who were less active, but also individuals reporting more than ten weekly hours of sports activities, had higher mortality compared to the reference group [27]. Moreover, prolonged endurance sports practice seems to be associated with an increased risk of atrial fibrillation [28]. We have previously reported that cumulative years of regular endurance exercise was associated with an increased risk of atrial fibrillation of 16% per decade in Norwegian middle-aged and older men [29]. The association is less studied in women, but rudimentary reports suggest an increased risk of atrial fibrillation related to exposure to endurance exercise over decades also among female athletes [30]. Lastly, sudden cardiac deaths occasionally seen in both young and middle-aged athletes have raised concerns as to whether high-intensity endurance sports promote severe cardiac conditions [31].

While prospective population-based health studies have provided important knowledge regarding the effects of moderate PA on various health outcomes, most studies have failed to capture the highest levels of PA, especially in older individuals who are upholding strenuous endurance exercise into older ages. The overall aim of The Birkebeiner Ageing Study is to complement previous studies by prospectively studying aging and health outcomes associated with prolonged exposure to endurance sport practice and other lifestyle habits in recreational athletes who are still participating in a long-distance cross-country ski competition when aged 65 years and older.

## Methods

### Study design

The Birkebeiner Ageing Study is a prospective cohort study among older recreational endurance athletes in Norway. The study is based on a questionnaire survey with periodical follow-up approximately every fifth year. The questionnaires used in the study address the participants’ history of endurance sport practice, current and previous lifestyle habits, including leisure-time PA, diseases and use of medications and mental and physical health status.

### Objectives and hypothesis

The overall objective of the study is to explore associations between endurance sport practice, leisure-time PA and other lifestyle habits with aging, disability and health outcomes in older recreational endurance athletes.

The primary objective is to examine the association between participation in endurance sports and risk of mortality and morbidity during long-term follow-up. The secondary objective is to describe physical, mental and overall health status related to aging, social and psychological factors, and gender differences in older recreational endurance athletes. The tertiary objective is to compare recreational endurance athletes and less physically active individuals of the same age.

We hypothesize that participation in endurance sports is associated with 1) low long-term mortality and morbidity, 2) preserved physical and mental health, and 3) that older recreational endurance athletes have reduced mortality, compression of morbidity, less disability and improved overall health status compared with less physically active individuals of the same age.

### Study population and eligibility criteria

All recreational skiers who participated in The Birkebeiner race in 2009 or 2010, had registered a postal address in Norway and were aged 65 years or older at that time, were invited to attend the baseline survey.

The Birkebeiner cross-country ski race is a long-distance endurance competition with a course of 54 km (www.birkebeiner.no/en). The course crosses three mountains, with a total of approximately 1,000 uphill meters and the race is known as one of the most challenging cross-country ski competitions worldwide. The Birkebeiner race was arranged for the first time in 1932 and has with few exceptions been arranged as an annual event ever since. Many skiers representing all parts of Norway and international participants complete the race year after year, with the highest number of attendees in a single race being almost 17,000 (2009).

The definition of an athlete by the American Heart Association is “one who participates in an organized team or individual sport that requires regular competition against others as a central component, places a high premium on excellence and achievement, and requires some form of systematic (and usually intense) training” [32]. Further, it has been suggested that a recreational athlete exercise for a minimum of four hours per week, with the intention to participate in competitions [33]. Cross-country skiing has been classified as an endurance sport with high intensity and high cardiovascular demand [14]. The long and steep course and often challenging weather conditions during the Birkebeiner race require that participants are well prepared, and thus, regular endurance exercise such as running, cycling and skiing, and high physical fitness could be considered mandatory to complete the race. The highly sought Birkebeiner medal is awarded skiers completing the race within the average time of the top five participants in each five-year age class plus 25%. Having achieved the Birkebeiner medal could be considered a proxy for the highest levels of physical fitness [34, 35].

### Baseline characteristics

Out of 658 invited skiers, 509 men and 46 women (84%) consented to participate and completed the baseline survey questionnaire during 2009 and 2010. Four were aged < 65 years and excluded. Compliance with filling out questionnaires was high, with missing data < 10% for all variables currently presented. An overview of baseline characteristics of the study participants, stratified by gender, is presented in Table 1. The mean age was 68.8 (median 68, range 65—90) years. In total, 482 (89%) of the participants reported living with a spouse and 231 (42%) were university educated.

**Table 1:** Characteristic of older recreational endurance athletes attending the baseline survey of the Birkebeiner Ageing Study in 2009–10

	**Men (*n* = 505)**	**Women (*n* = 46)**
	**Mean (median, IQR)**	
Age	68.9 (68, 5)	67.5 (67, 3)
Height	178.5 (178, 7)	165.0 (165, 5)
Weight	75.4 (75, 10)	59.9 (60, 7)
Body mass index	23.6 (23.5, 2.5)	22.0 (22.0, 2.4)
Years of ski race participation	17.0 (14, 20)	12.0 (9, 17)
	**% (n)**	
Living with spouse	89.2(445)	82.2 (37)
Education		
Primary/Secondary school	16.6 (83)	8.9 (4)
High school	42.2 (211)	35.6 (16)
University	41.2 (206)	55.6 (25)
Smoking status		
Never	61.3 (309)	75.6 (34)
Previous	37.9 (191)	24.4 (11)
Current	0.8 (4)	0.0 (0)
Years of regular endurance exercise		
Less than 10	5.8 (28)	9.5 (4)
10–29	32.3 (156)	52.4 (22)
30–49	37.7 (182)	28.6 (12)
50 or more	24.2 (117)	9.5 (4)
Physical activity past 12 Months		
Inactive	0.2 (1)	0.0 (0)
Light	9.8 (48)	14.3 (6)
Moderate	51.0 (251)	54.8 (23)
Vigorous	39.0 (192)	31.0 (13)
Current or previous use of		
Blood pressure medication	15.7 (76)	21.4 (9)
Lipid lowering drugs	15.0 (73)	14.6 (6)
Cardiovascular conditions		
Coronary heart disease	3.6 (18)	2.3 (1)
Stroke	1.6 (8)	0.0 (0)
Atrial fibrillation	13.8 (69)	8.7 (4)
Diabetes	0.8 (4)	2.4 (1)

Continuous variables are presented as mean, median and interquartile range (IQR). Categorical variables are given in percentages and as numerical values

The mean number of years of Birkebeiner race participation was 16.6. The skiers reported an average of 33.4 years of regular endurance exercise, and almost one out of four reported that they had practiced endurance exercise regularly for more than 50 years. The vast majority of the participants reported that they had upheld moderate (51%) or high (38%) levels of PA during the past 12 months before baseline.

Few athletes reported current or previous use of lipid-lowering drugs (15%) and blood pressure-lowering medication (16%) and only four (1%) were current smokers. The prevalence of coronary heart disease (3%), diabetes (1%) and stroke (1%) was very low.

### Questionnaires

The baseline survey questionnaire was designed based on questionnaires used in large health surveys in Norway previously, and included in the Cohort of Norway (CONOR) [36]. In particular, with the aim to allow comparison with a non-athletic general population, we chose questions used as part of the population-based Tromsø study [37]. In addition, we included single questions addressing participation in endurance sports and various matters, as well as established and validated instruments for assessments of leisure-time PA, mental, physical and overall health status, self-esteem and mastery. The original questionnaires and an English translation of the baseline questionnaire are published online [38]. Height and weight are self-reported, and body mass index is calculated as weight (kg) divided by squared height (m).

Follow-up surveys contain the majority of the same questions and instruments as the baseline survey. In 2014, also information on risk factors for osteoporosis and results of bone mass density measurements were included for the women.

#### Leisure-time physical activity and endurance sports practice

We assess leisure-time PA during the past 12 months with the question “How physically active have you been during the past year in your leisure time. If your activity level varies between summer and winter, note an average value. Tick one only.” The four levels of activity: (1) sedentary (reading, watching television, other sedentary activity); (2) light PA (walking, cycling or other activity for at least 4 h per week); (3) moderate PA (light sports, heavy gardening, for at least 4 h per week); and (4) high PA (regular hard exercise or competitive sports several times per week). The question is well validated in previous studies and correlates with cardiorespiratory fitness as examined by maximal oxygen consumption [39–41]. Endurance sport practice is assessed using unvalidated questions about the history of regular endurance exercise and participation in the Birkebeiner race and other endurance competitions. In particular, we ask “How many times have you completed the Birkebeiner race?”, and “How many times have you been awarded with the Birkebeiner medal?”.

#### Morbidity and use of medication

With the aim to study illness, injuries and chronic diseases, we ask “Have you/have you ever had [condition]”. The questionnaires contain questions regarding cerebral stroke, myocardial infarction, angina pectoris, atrial fibrillation, diabetes mellitus, cancer, chronic obstructive pulmonary disease and lung emphysema, asthma, osteoporosis, fractures, fibromyalgia, chronic pain, and injury requiring hospital admission. We also ask “Have you during the last year suffered from pain and/or stiffness in muscles and joints that have lasted for at least three months?”.

We ask the participants to report current and previous use of medications for high blood pressure, lipid-lowering medications, analgetics, sleeping pills, tranquilizers, antidepressants, allergy pills, asthma medication and dietary supplements. Women are asked to report use of hormones.

#### Physical, mental and overall health status

We use the Norwegian translation of the 12-item short-form healthy survey (SF-12) to assess the participant's overall physical and mental health status. The instrument is validated in older people and measures physical functioning, role physical, bodily pain, general health, vitality, social functioning, role emotional and mental health, and provides weighted sumscores for physical and mental health status, respectively [42].

SF-12 has shown a high degree of correspondence with the 36-item short-form (SF-36) and the shorter SF-12 is more efficient for use as part of comprehensive health surveys [43–45].

The Modified Health Assessment Questionnaire (MHAQ) assesses physical function with eight questions about physical abilities during activities of daily living. Each question is scored on a 4-level scale. The instruments were originally developed for use in individuals with musculoskeletal disorders and aim to assess perceived patient satisfaction regarding activities of daily living [46]. We used the Norwegian translation of MHAQ in this study.

#### Psychological factors

The ability to adapt and cope is assessed with the Pearlin Mastery Scale, and global self-worth is assessed with the Rosenberg self-esteem scale [47, 48]. In addition to these validated standard instruments, participants are asked to score their personal relation with sports and motivation on a four-level scale ranging from “Strongly disagree” to “Strongly agree” for three items: 1) “The sport means a lot to my quality of life”, 2) “Good sports performance means a lot to me”, and 3) “Participation in the Birkebeiner cross-country ski race is a motivation for practicing systematic exercise”. We ask “Do you have or have you ever had psychological problems for which you have sought help?” and of reports of if the participants have felt nervous or worried, anxious, confident and calm, irritable, happy/optimistic, down/depressed and lonely during the last two weeks, on a 4-level scale.

#### Socioeconomic status and other lifestyle habits

We use CONOR-questions to assess the highest level and total years of education completed, type of dwelling, marital status, current occupational situation and social activities. Habits of smoking, alcohol consumption, and coffee and tea drinking are assessed in detail with questions used in CONOR.

#### Mortality

Mortality data were collected from the Norwegian population register, which is available online from Norwegian hospitals.

### Comparison with non-athletic cohorts

The questionnaires applied in the present study population are either identical or similar to questionnaires applied in the population-based Tromsø study, allowing comparison with non-athletic people representative of the general population. The Tromsø study is a prospective cohort study initiated in 1974, aiming to study health outcomes in a general population. Up to date, seven surveys have been completed, including the sixth and seventh survey in 2007/2008 and 2015/2016, respectively. The study is considered representative of a Norwegian Caucasian population [49]. The sixth survey was conducted at approximately the same time as the baseline survey of the Birkebeiner Ageing Study and is the most suitable for comparison: A total of 6098 individuals aged 65 years and older were invited to the sixth survey, 3341 women and 2757 men. Out of these, 2150 women (64%) and 1867 men (68%) participated [50].

### Statistical considerations

Analysis of descriptive data between groups may be tested by the Mann–Whitney U test for continuous data and with the Pearson χ^2^ test for categorical data. We will apply directed acyclic graphs for a priori model selection. Although the analytical method is likely to vary by the research question at hand, we suggest that regression methods are suitable for the prospective design of this study. For prospective analyses of self-reported outcomes logistic models may be applied. However, for outcomes with a high prevalence and where relative risk estimates are preferred over odds ratios, log-Poisson models with robust error variance may be preferred [51]. For time-to-event data, survival analysis by Cox proportional hazards regression will be applied in order to present hazard ratios and corresponding 95% confidence intervals. Model assumptions for the Cox model may be assessed by Schoenfeld’s test of residuals and log minus log plots. Furthermore, unadjusted Kaplan–Meier curves as well as adjusted survival curves may be presented.

## Discussion

This prospective study, with long-term follow-up, will provide data on associations between life-long endurance sport participation, aging and health outcomes in a cohort of older recreational athletes.

A main strength of the study is that it has the potential to complement previous population-based health surveys, with knowledge regarding long-term health outcomes in the most active part of the older population. The cohort in this study is characterized by an average exposure to strenuous endurance sport practice, including repeated participation in a long-distance arduous cross-country ski race, of several decades. Furthermore, at baseline the study population was remarkably healthy, with low prevalence of both cardiovascular risk factors and cardiovascular conditions. As we are not aware of any other prospective studies of recreational older endurance athletes with such a long follow-up, the prospective study design and planned long-term follow-up of at least 20 years are further main assets of the study and could potentially provide novel insights. The high participation rate in the baseline survey and high compliance with completing the baseline questionnaire correctly, promise high-quality self-reported data.

The main limitation of the study is that it is entirely based on self-reporting by the study participants that could be affected by both over- and underestimation of lifestyle habits and diseases, and recall bias in reports of long-term exposure to exercise. The low number of female participants in the study is another limitation.

### Study status

A research paper addressing the association between leisure-time PA, endurance sport practice and the risk of atrial fibrillation in older men, based on data from the baseline survey and data from the sixth survey of the Tromsø study, was published in 2014 [52]. Two substudies, including 30 male and 24 female athletes, respectively, have been previously published; one study investigating orthostatic intolerance in older male athletes and controls [53], and one study on the association between PA and bone mineral density in older female athletes and a control group from the Tromsø study [54]. The first research paper using data from the longitudinal follow-up surveys in the study, addressing the cumulative prevalence and risk of atrial fibrillation and stroke related to exercise and sport practice, was published recently [55]. Selected results from the study have also been presented to the study participants in periodically distributed newsletters.

Further follow-up surveys with questionnaires are planned for 2024 and 2029 when the youngest participants in the study will have reached the age of 85 years. We also intend to invite new participants from the Birkebeiner race or other endurance competitions, with the aim of increasing the study size. In particular, we will strive to reduce the underrepresentation of women and prioritize recruitment of new cohorts of female athletes [56].

### User involvement

A user interest group consisting of five study participants (two women, three men), together with the first and last author, have been involved in the planning and execution of the study from its very beginning. The researchers arranged five meetings with the user interest group during the period 2009 to 2014.

## Data Availability

The datasets generated in the current study are not publicly available because the study is not yet completed, but may be available from the corresponding author in the future on reasonable request.

## Abbreviations

PA: Physical activity
CONOR: Cohort of Norway
SF-12: Short-form 12
SF-36: Short-form 36
MHAQ: Modified Health Assessment Questionnaire
